# Topoisomerase I copy number alterations as biomarker for irinotecan efficacy in metastatic colorectal cancer

**DOI:** 10.1186/s12885-016-3001-y

**Published:** 2017-01-11

**Authors:** Jesper Andreas Palshof, Estrid Vilma Solyom Høgdall, Tim Svenstrup Poulsen, Dorte Linnemann, Benny Vittrup Jensen, Per Pfeiffer, Line Schmidt Tarpgaard, Nils Brünner, Jan Stenvang, Mette Yilmaz, Dorte Lisbet Nielsen

**Affiliations:** 1 Department of Oncology, Herlev Hospital, University of Copenhagen, Herlev Ringvej 75, DK-2730 Herlev, Denmark; 2 Department of Pathology, Herlev Hospital, University of Copenhagen, Herlev Ringvej 75, DK-2730 Herlev, Denmark; 3 Department of Oncology, Odense University Hospital, Sdr. Boulevard 29, 5000 Odense C, Denmark; 4 Faculty of Health and Medical Sciences, Department of Veterinary Disease Biology, Section for Molecular Disease Biology and Sino-Danish Breast Cancer Research Centre, University of Copenhagen, Copenhagen, Denmark; 5 Department of Oncology, Aalborg University Hospital, Hobrovej 18-22, 9100 Aalborg, Denmark

**Keywords:** Biomarker, Colorectal cancer, FISH, Gene copy number, Irinotecan, Topoisomerase I

## Abstract

**Background:**

No biomarker exists to guide the optimal choice of chemotherapy for patients with metastatic colorectal cancer. We examined the copy numbers (CN) of topoisomerase I (*TOP1*) as well as the ratios of *TOP1*/CEN-20 and *TOP1*/CEN-2 as biomarkers for irinotecan efficacy in patients with metastatic colorectal cancer.

**Methods:**

From a national cohort, we identified 163 patients treated every third week with irinotecan 350 mg/m^2^ as second-line therapy. Among these 108 were eligible for analyses and thus entered the study. Primary tumors samples were collected and tissue microarray (TMA) blocks were produced. FISH analysis was performed using two probe-mixes: *TOP1*/CEN-20 and *TOP1*/CEN-2. Only samples harboring all three signals (*TOP1*, CEN-20 and CEN-2) using FISH were included in the analyses.

**Results:**

In the *TOP1*/CEN-20 probe-mix the median *TOP1*- and CEN-20 CN were 4.46 (range: 1.5–9.5) and 2.00 (range: 0.55–4.55), respectively. The median *TOP1*- and CEN-2 CN in the *TOP1*/CEN-2 probe-mix, were 4.57 (range: 1.82–10.43) and 1.98 (range: 1.22–6.14), respectively. The median *TOP1*/CEN-20 ratio and *TOP1*/CEN-2 ratio were 1.25 (range: 0.92–2.90) and 2.05 (range: 1.00–6.00), respectively. None of the markers *TOP1* CN, *TOP1*/CEN-20-ratio or *TOP1*/CEN-2-ratio were associated with progression free survival, overall survival or baseline characteristics. Yet, we observed a borderline association for a stepwise increase of the *TOP1* CN in relation to objective response as hazard ratio were 1.35 (95% CI 0.96–1.90; *p* = 0.081).

**Conclusions:**

We verified a borderline significant association between increasing *TOP1* CN and objective response as previously reported. Applying the probes representing CEN-20 and CEN-2, in order to investigate the ratios of *TOP1*/CEN-20 and *TOP1*/CEN-2 provided no further information in search of a biomarker driven patient stratification. Other biomarkers to be paired with *TOP1* CN are therefore highly warranted.

## Background

Colorectal cancer (CRC) is the third most common cancer and the fourth most common cause of cancer death worldwide [1]. Almost 50% of patients diagnosed with CRC will develop metastatic disease [2]. Standard of care for patients with non-resectable metastatic colorectal cancer (mCRC) is combination chemotherapy with 5-fluorouracil (5-FU)/leucovorin (LV)/oxaliplatin (FOLFOX) or 5-FU/LV/irinotecan (FOLFIRI) with or without a targeted agent [3]. In first-line therapy, FOLFIRI and FOLFOX are considered equally effective [4].

Predictive biomarkers for the efficacy of 5-FU, irinotecan and oxaliplatin have been suggested but none, so far, have been implemented in the clinical setting [5]. However, a significant fraction of the patients does not benefit from the treatment but may experience serious side effects only. The topoisomerase 1 (Top1) protein is an essential nuclear enzyme for vital cellular processes such as DNA replication, transcription, translation, recombination and repair. The primary function of Top1 is to unwind and uncoil the supercoiled DNA double helix by transiently cleaving one of the two strands and thereby allowing its rotation over the other strand [6]. This intermediate cleavage state is termed the Top1 cleavage complex (Top1cc). Top1 is the target of irinotecan (CPT-11), a camptothecin derivative, which is metabolized to the active metabolite SN-38 which binds to and stabilizes the Top1cc, whereby the rapidly moving DNA replication and transcription complexes collide with this trapped Top1ccs. The main cytotoxicity induced by irinotecan is caused by DNA double-strand breaks during DNA replication and the presence of Top1 is thus a prerequisite for this cytotoxic effect [7, 8].

The plausible link between tumor tissue levels of Top1 and effect of Top1 inhibitors in cancer treatment [9] has been investigated by different methods. In vitro studies with colon cancer cell lines have demonstrated a significant correlation between the *topoisomerase I gene* (*TOP1*) copy numbers (CN) or Top1 protein expression and the sensitivity to SN-38 [9, 10]. A prospective clinical trial (FOCUS) investigated the association between Top1 protein expression and benefit from FOLFOX and FOLFIRI in patients with mCRC [11]. A significant association between Top1 protein immunoreactivity and clinical benefit from FOLFIRI or FOLFOX was found. However, conflicting results have been reported since the findings could not be validated in a subsequent study from the same group (FOCUS3) [12]. Furthermore, the results from another large prospective trial (CAIRO) showed no correlation of Top1 immunoreactivity and response to irinotecan in patients with mCRC [13]. An explanation to these diverse results was recently provided by (Maughan et al.) who showed that the antibody used for IHC in the above mentioned studies did not result in reproducible staining patterns [12]. We have recently introduced another approach for Top1 quantitation in cancer cells. Instead of immunohistochemistry IHC to quantitate protein expression we used fluorescence in situ hybridization (FISH) to assess *TOP1* gene copy number (CN) status as a proxy for the overall Top1 protein levels. We have previously identified a significant correlation between the *TOP1* gene CN, *TOP1* mRNA expression and Top1 protein levels using data generated from in vitro studies on CRC cell lines [14].

The (*TOP1*) gene is located on chromosome 20 at 20q12 and this region frequently undergoes CN alterations in various cancers [14–17]. In CRC, the *TOP1* aberration has been reported by applying a *TOP1*/CEN-20 fluorescence in situ hybridization (FISH) probe-mix. The *TOP1* CN gain in CRC has been reported to be in the range of 53–84%, whereas *TOP1*/CEN-20 ratios ≥ 1.5 or ≥2.0 were in the range of 30–40% and 10–20%, respectively [14, 18, 19]. Current data suggests that *TOP1* CN increases occur predominately in conjunction with the rest of 20q [14, 16, 17, 20] and the CEN-20 region [14, 18]. Therefore the usage of the *TOP1*/CEN-20 ratio may underestimate the genuine *TOP1* amplifications. Chromosome 2 (CEN-2) has been found to be the least affected by independent numeric aberrations in the genome, and has therefore been combined with *TOP1* in a *TOP1*/CEN-2 probe-mix to distinguish between *TOP1* gene gain and genuine *TOP1* amplifications [21]. These two different types of CN alterations have been demonstrated to have differential prognostic effects in stage III CRC patients [21]. In a metastatic setting a borderline significant association (*p* = 0.07) between an increase in *TOP1* CN and objective response to second-line treatment with irinotecan monotherapy has been reported [19].

Therefore, we applied both a *TOP1*/CEN-20 and a *TOP1*/CEN-2 FISH probe-mix to 108 tumors from mCRC with the aim to investigate *TOP1* CN and the ratios of both *TOP1*/CEN-20 and *TOP1*/CEN-2 as biomarkers for irinotecan efficacy.

## Methods

The patients included in this explorative study were extracted from a national cohort of 498 patients with mCRC who all received irinotecan in combination with the epidermal growth factor receptor inhibitor, cetuximab as third-line treatment from 1st of January 2005 to 1st of August 2008 at the Departments of Oncology at Herlev, Odense, and Aalborg Hospitals in Denmark. The inclusion period for the national cohort was specifically selected because it preceded the introduction of KRAS testing. Therefore, mutational status for KRAS, NRAS and BRAF is unknown in this cohort. From this cohort we identified 163 consecutive patients who were treated every third week with irinotecan 350 mg/m^2^ as second-line therapy. Disease evaluation was performed during treatment using computed tomography (CT) scans of the thorax and abdomen every 9–12 weeks to evaluate response according to the RECIST 1.0 criteria [22]. Data for objective response, progression-free survival (PFS) and overall survival (OS) were extracted from the database. PFS was defined as time from start of treatment to progression or death from any cause. OS was defined as time from start of treatment to death from any cause. Last follow-up on survival was done in October 2014. The study was approved by the Research Ethics Committee of Copenhagen (H-KA-20060094). Reporting of the results was prepared according to the REMARK criteria [23].

### Tumor material

Only primary tumor samples were used. Formalin-fixed paraffin-embedded samples from either resections or core needle biopsies were collected. The presence of tumor cells in the samples was confirmed by a pathologic review performed by JP and an experienced gastro-intestinal pathologist (DL). Tissue microarray (TMA) blocks were produced; each containing tumor material from 18 patients with two 1 mm tissue cores per patient sample. Standard procedures were used for preparation of the TMA blocks. The TMA blocks were cut in 2-μm sections and stored at 5 °C until hybridization.

### Fluorescence in situ hybridization (FISH)

The probes for *TOP1*, CEN-20 and CEN-2 were developed and produced by the Department of Pathology, Herlev University Hospital. All probes were sequenced to confirm that all base pairs exactly matched the *TOP1* gene and the centromeres CEN-20 and CEN-2. Two probe-mixes: *TOP1*/CEN-20 and *TOP1*/CEN-2 were produced. The probes were labelled with Texas red (*TOP1*) and fluorescein isothiocyanate (FITC), green for CEN-20 and CEN-2. Only samples harboring all three signals (*TOP1*, CEN-20 and CEN-2) using FISH were included in the analyses. Since the *TOP1* probe was present in both probe-mixes, it was counted twice - independently.

Two slides from each TMA block were deparaffinized, rehydrated, boiled in pre-treatment buffer for 10 min and cooled in the buffer for 15 min at room temperature followed by 2 × 3 min in wash buffer (1:20) (K5799 - Dako). RTU-pepsin was added for 2 min at 37 °C and removed in wash buffer for 2 × 3 min. Following ethanol (70% → 96% → 99%) dehydration and 15 min. air-dry, 10 μL of *TOP1*/CEN-20 probe was applied to the center of one of the two slides and 10 μL of *TOP1*/CEN-2 probe-mix was applied to the other slide. Non-specific binding of probe was removed by stringency wash (1:20) at 65 °C for 10 min (K5731 - Dako). A fluorescence microscope (Olympus BX61) with DAPI, FITC, Texas Red and dual FITC/Texas Red filter was used for visualization of the signals. Signal counting was performed by JP, blinded to all patient data. In case of ambiguity, a senior pathologist (DL) was consulted. A minimum of sixty *TOP1* signals in total, 30 from each of the two cores, were counted in non-overlapping cancer nuclei with well-defined morphology and distinct fluorescent signals. If the fluorescent intensity was weak or insufficient tumor tissue was present, a new section was cut. If signals continued to be too weak for clear interpretation, the sample was excluded from the analyses.

### Cutoffs and Definitions

A cutoff of 2 for the ratios of *TOP1*/CEN-20 and *TOP1*/CEN-2 were used in this study.

Tumors were classified as “amplified” when (*TOP1*/CEN-20 ratio ≥2 and *TOP1*/CEN-2 ratio ≥2) and as “polysomies” when (*TOP1*/CEN-20 ratio ≤2 and *TOP1*/CEN-2 ratio ≥2).

### Statistics

To examine the association between *TOP1* CN from the two probe-mixes, a Reliability Analysis with an Intraclass correlation was performed. Pearson’s chi-squared test was used to test for associations between baseline characteristics and *TOP1* CN and *TOP1*-/CEN-20- and CEN-2 ratios. The baseline characteristics were: gender, age, WHO performance status (PS), location of primary tumor, resection of primary tumor, number of metastatic sites, prior chemo- and radiotherapy, and presence of lung or liver metastases. The *TOP1* CN per cell was divided by the median value into two groups. *TOP1*/CEN-20- and CEN-2 ratios were divided into ≥2 and <2. *p*-values and 95% confidence intervals (CI) are reported.

The final analyses of survival data were performed in October 2014. Cox proportional hazards (CPH) regression models were used to test for association between time-to-event endpoints and both a dichotomized value and a continuous value of *TOP1* signal count, *TOP1*/CEN-20 ratio, and *TOP1*/CEN-2 ratio, with adjustment for baseline characteristics. We also used CPH models to test “amplified” (*TOP1*/CEN-20 ratio ≥2 and *TOP1*/CEN-2 ratio ≥2) vs “non- amplified” tumors and “polysomies” (*TOP1*/CEN-20 ratio ≤2 and *TOP1*/CEN-2 ratio ≥2) vs “non-polysomies”. Wald chi-squared *p*-values hazard ratio (HR) estimates with estimated 95% CI interval were reported.

The association between *TOP1* CN, *TOP1*/CEN-20 ratio, and *TOP1*/CEN-2 ratio and objective response rate was tested using logistic regression. Responders were defined as patients with complete (CR) or partial response (PR) according to RECIST 1.0 criteria. We also performed the same analysis using clinical benefit rate (CBR). Clinical benefit was defined as CR, PR or stable disease (SD) ≥6 months. For these tests, Wald chi-squared *p*-values and odds ratio (OR) estimates with 95% CI were reported.

## Results

**Fig. 1:**
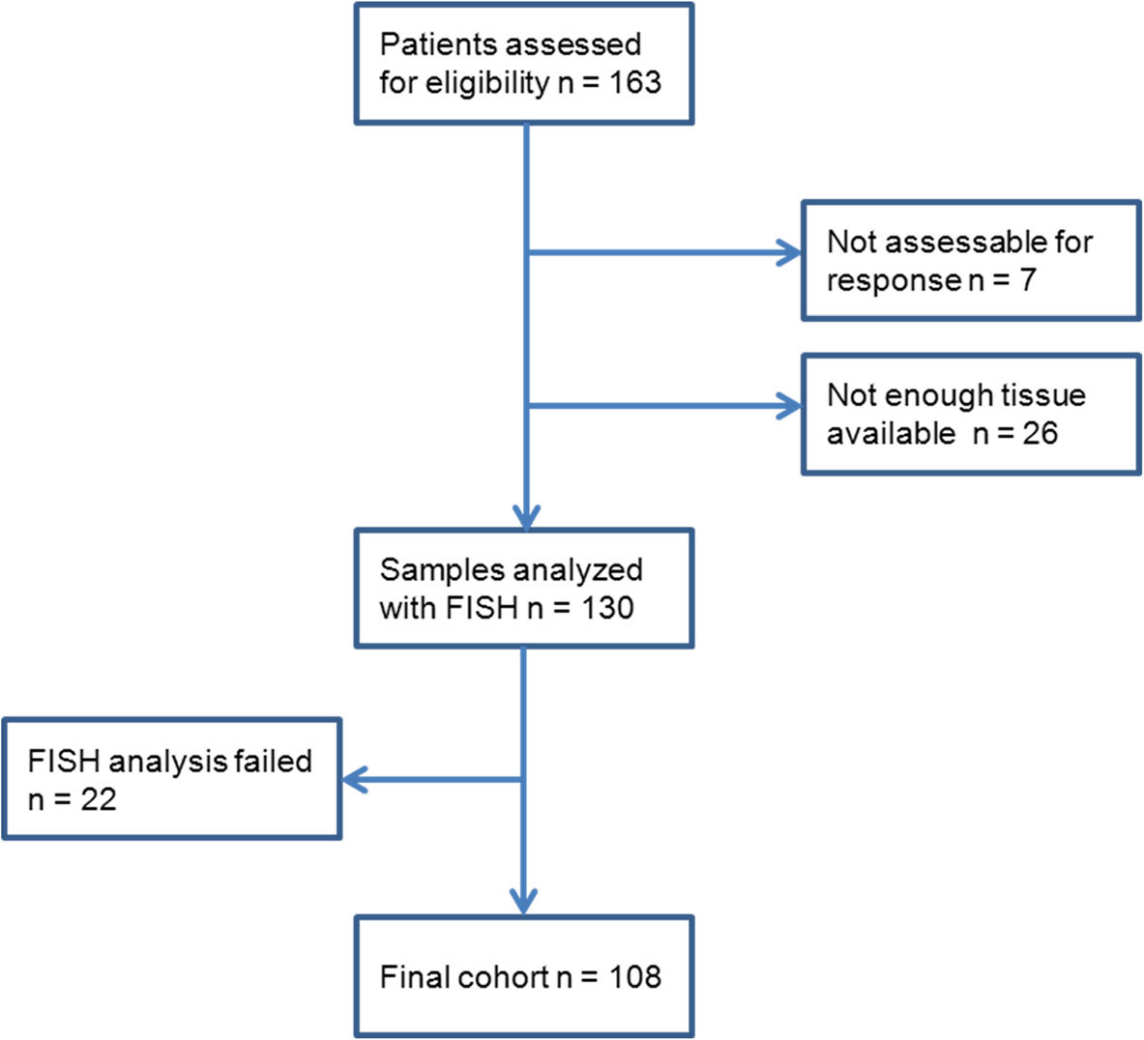
CONSORT diagram showing the flow of patients and samples

In the database of our national cohort, we searched for patients having received irinotecan monotherapy as second-line therapy. We identified 163 patients. Of the 163, 26 (16%) patients were excluded as they did not have enough tissue available for analyses, in 22 (13%) we failed to produce countable FISH signals from both probe-mix (*TOP1*/CEN-20 and *TOP1*/CEN-2) after two attempts and 7 patients were not assessable for response due to either concurrent surgery or radiotherapy. Thus, 108 patients were eligible for analyses in the study (Fig. 1).

*TOP1* CN was counted twice due to the use of two probe-mixes. When comparing the results of *TOP1* CN from the two probe-mixes, the Single Measures Intraclass correlation was r = 0.74 (CI 0.64–0.82; *p* <0.001). The Spearman correlation between *TOP1* and CEN-2 was: r = 0.44 (*p* <0.001), between *TOP1* and CEN-20: r = 0.82 (*p* <0.001) and between CEN-2 and CEN-20: r = 0.41 (*p* <0.001).

**Fig. 2:**
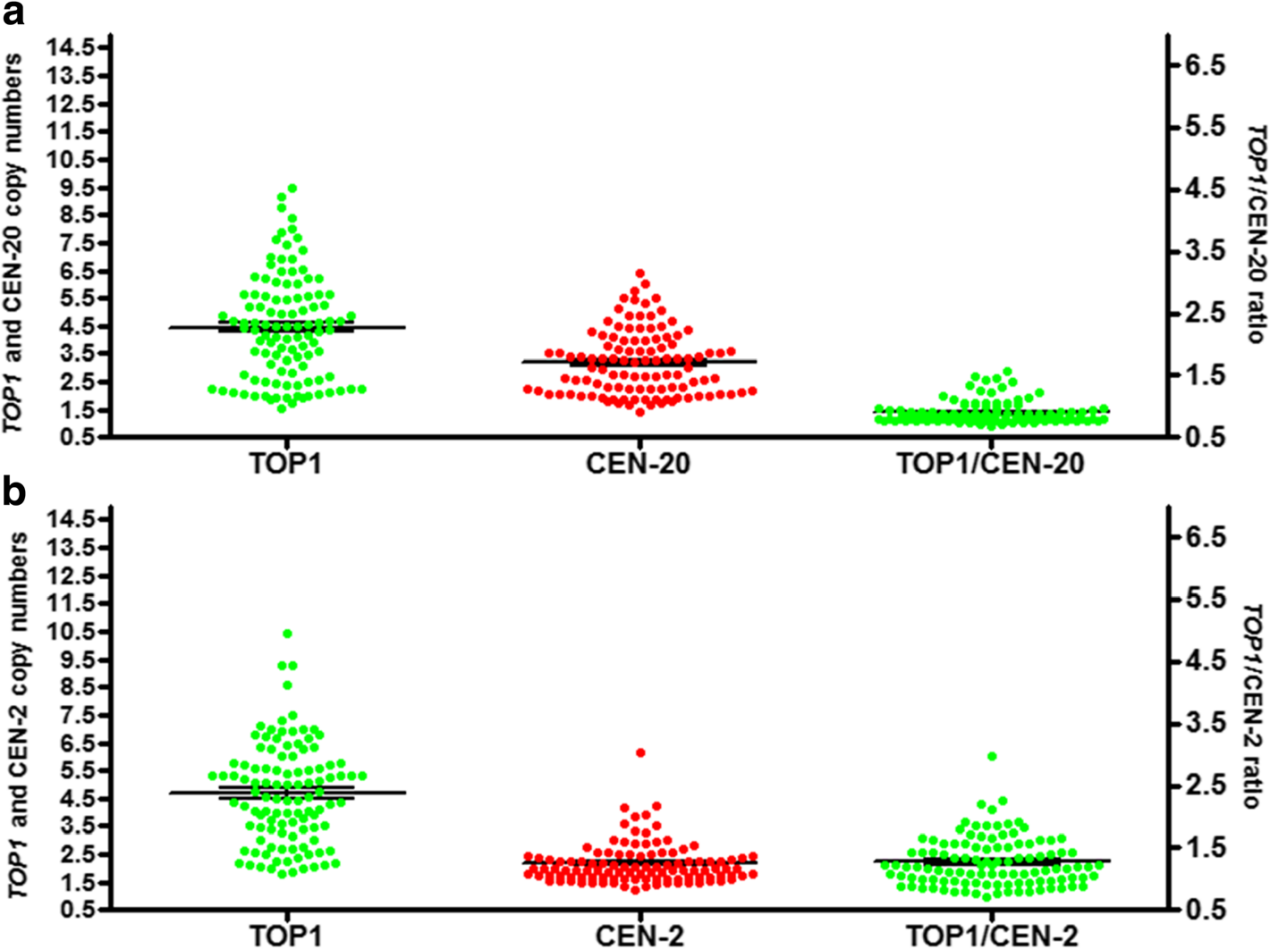
Distribution of *TOP1*, CEN-20 and CEN-2 copy numbers and of *TOP1*/CEN-20 and *TOP1*/CEN-2 ratios. Distribution in colorectal cancer samples determined by FISH. **a** Showing the distribution of the copy numbers for probe-mix *TOP1*/CEN-20. **b** Showing the distribution of the copy numbers for probe-mix *TOP1*/CEN-2

For the *TOP1*/CEN-20 probe-mix, the median *TOP1*- and CEN-20 CN were 4.46 (range: 1.5–9.5) and 2.00 (range: 0.55–4.55), respectively. The median *TOP1*- and CEN-2 CN in the *TOP1*/CEN-2 probe-mix, were 4.57 (range: 1.82–10.43) and 1.98 (range: 1.22–6.14), respectively. The median *TOP1*/CEN-20 ratio and *TOP1*/CEN-2 ratio were 1.25 (range: 0.92–2.90) and 2.05 (range: 1.00–6.00), respectively (Fig. 2). The distribution of *TOP1* CN and ratios for the 108 patients are shown in Table 1. We used the median *TOP1* CN (probe-mix *TOP1*/CEN-20) to test the association with baseline characteristics (Table 2). Significant associations between prior chemotherapy and *TOP1*/CEN-2 ratio and between liver metastases and *TOP1*/CEN-20 ratio were found. However, these results were not significant after correction for multiple testing (data not shown).

**Table 1:** Distribution of *TOP1* CN and ratios for the 108 patients

	*TOP1* CN No (%)	*TOP1*/CEN-2 ratio No (%)	*TOP1*/CEN-20 ratio No (%)
<2	7 (6)	50 (46)	98 (91)
2.0 – 2.99	23 (21)	36 (33)	10 (9)
3.0 – 3.99	14 (13)	18 (17)	-
4.0 – 4.99	25 (23)	3 (3)	-
5.0 – 5.99	15 (14)	-	-
6.0 – 6.99	13 (12)	1 (1)	-
>7	11 (10)	-	-

**Table 2:** Baseline Characteristics and *TOP1* copy number

		*Total N* (%)	TOP1 CN per cell	TOP1 CN per cell	Pearson Chi-Square test p	TOP1/CEN-20 ratio ≥ 2 N (%)	TOP1/CEN-20 ratio < 2 N (%)	Pearson Chi-Square test p	TOP1/CEN-2 ratio ≥ 2 N (%)	TOP1/CEN-2 ratio < 2 N (%)	Pearson Chi-Square test p
			>median 4.46 No (%)	≤ median 4.46 No (%)							
Patients included		*108 (100)*	54 (50)	54 (50)		10 (9)	98 (91)		58 (54)	50 (46)	
Gender	Males	65 (60)	35 (54)	30 (46)	0.33	6 (9)	59 (91)	0.99	37 (57)	28 (43)	0.41
	Females	43 (40)	19 (44)	24 (56)		4 (9)	39 (91)		21 (49)	22 (51)	
Age	≥65	52	23 (44)	29 (56)	0.25	4 (8)	48 (92)	0.59	26 (50)	26 (50)	0.46
	<65	56	31 (55)	25 (45)		6 (11)	50 (89)		32 (57)	24 (43)	
WHO PS	0	51 (47)	25 (49)	26 (51)	0.99	5 (10)	46 (90)	0.95	25	26	0.33
	1–2	53 (49)	26 (49)	27 (51)		5 (9)	48 (91)		31	22	
	unknown^a^	4 (4)									
Location primary tumor	Right	24 (22)	13 (54)	11 (46)	0.82	1 (4)	23 (96)	0.36	10 (42)	14 (58)	0.32
	Left	43 (40)	22 (51)	21 (49)		6 (14)	37 (86)		23 (53)	20 (47)	
	Rectum	41 (38)	19 (46)	22 (54)		3 (7)	38 (93)		25 (61)	16 (39)	
Primary tumor resected	Yes	98 (91)	48 (49)	50 (51)	0.51	7 (7)	91 (93)	0.02	51 (52)	47 (48)	0.28
	No	10 (9)	6 (60)	4 (40)		3 (30)	7 (70)		7 (70)	3 (30)	
Number of metastatic sites	1	37 (34)	17 (46)	20 (54)	0.78	3 (8)	34 (92)	0.87	22 (59)	15 (41)	0.14
	2	35 (33)	19 (54)	16 (46)		4 (11)	31 (89)		14 (40)	21 (60)	
	>2	36 (33)	18 (50)	18 (50)		3 (8)	33 (92)		22 (61)	14 (39)	
*Prior chemotherapy*	F	10 (9)	7 (70)	3 (30)	0.10	3 (30)	7 (70)	0.054	9 (90)	1 (10)	0.01
	F + Oxa	95 (88)	47 (49)	48 (51)		7 (7)	88 (93)		49 (52)	46 (48)	
	F + Oxa + Bev	3 (3)	0 (0)	3 (100)		0 (0)	3 (100)		0 (0)	3 (100)	
Prior radiotherapy	Yes	5 (5)	2 (40)	3 (60)	0.65	0 (0)	5 (100)	0.47	4 (80)	1 (20)	0.23
	No	103 (95)	52 (50)	51 (50)		10 (10)	93 (90)		54 (52)	49 (48)	
Liver metastases	Yes	80 (74)	43 (54)	37 (46)	0.19	8 (10)	72 (90)	0.65	43 (54)	37 (46)	0.99
	No	28 (26)	11 (39)	17 (61)		2 (7)	26 (93)		15 (54)	13 (46)	
Lung metastases	Yes	46 (43)	25 (54)	21 (46)	0.44	3 (7)	43 (93)	0.40	24 (52)	22 (48)	0.78
	No	62 (57)	29 (47)	33 (53)		7 (11)	55 (89)		34 (55)	28 (45)	

*Abbreviations*: *CN* Copy number, *F* Fluorouracil (5-FU), *Oxa* Oxaliplatin, *Bev* Bevacizumab, *HR* Hazard ratio, *CI* Confidence interval

**Fig. 3:**
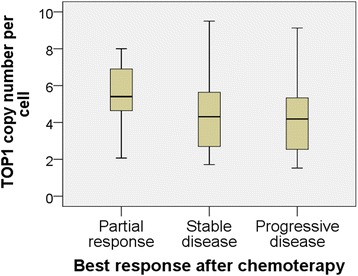
Boxplot. Distribution of *TOP1* copy number as a function of best response to chemotherapy. The top and bottom of the box represents the upper and lower quartiles and the black line in the box the median. The whiskers represent the maximum and minimum values, excluding outliers

Ten patients (9%) had PR, 46 (43%) had SD, and 51 (47%) had PD as best response. The distribution of *TOP1* CN for patients having PR, SD and PD is illustrated in Fig. 3. The OR estimates for a stepwise increase of the *TOP1* CN and *TOP1*/CEN-20 and *TOP1*/CEN-2 ratios in relation to objective response were 1.35 (CI 0.96–1.90; *p* = 0.081), 1.99 (CI 0.51–7.75; *p* = 0.32), and 1.34 (CI 0.69–2.64; *p* = 0.40), respectively. No significant association was found between CBR and a stepwise increase of the *TOP1* CN, *TOP1*/CEN-20- or *TOP1*/CEN-2-ratio as the OR estimates were 1.01 (CI 0.81–1.25; *p* = 0.94), 0.72 (CI 0.24–2.14; *p* = 0.56), and 0.88 (CI 0.54–1.42; *p* = 0.60), respectively.

The median PFS and OS were 3.8 months (range: 1.3–13.1) and 16.4 months (range: 4.6–91.6), respectively. None of the biomarkers *TOP1* CN, *TOP1*/CEN-20-ratio or *TOP1*/CEN-2-ratio when tested as continuous variables, were associated with PFS as HRs were 0.99 (CI 0.90–1.10; *p* = 0.88), 0.99 (CI 0.56–1.74; *p* = 0.97), and 1.10 (CI 0.85–1.35; *p* = 0.58), respectively. For OS, HRs were 0.98 (CI 0.89–1.08; *p* = 0.72), 1.02 (CI 0.61–1.70; *p* = 0.95), and 1.00 (CI 0.79–1.27; *p* = 0.98) for *TOP1* CN, *TOP1*/CEN-20-ratio and *TOP1*/CEN-2-ratio, respectively (Table 3).

**Table 3:** Univariate survival analyses

		Progression-free survival			Overall survival		
		*HR*	95% CI	p	HR	95% CI	p
Gender	Females	(ref)			(ref)		0.03
	Males	0.65	0.43–0.96	0.03	0.64	0.43–0.94	
Age	Per 1 year increase	0.99	0.97–1.02	0.63	0.98	0.96–1.01	0.16
WHO PS	0	0.84	0.57–1.24	0.38	0.59	0.39–0.88	0.009
	1–2	(ref)			(ref)		
	unknown^a^						
Location primary tumor	Right	0.74	0.45–1.23	0.25	1.21	0.72–2.02	0.48
	Left	0.87	0.57–1.35	0.54	1.14	0.74–1.76	0.55
	Rectum	(ref)			(ref)		
Primary tumor resected	Yes	0.78	0.41–1.51	0.46	0.78	0.40–1.50	0.49
	No	(ref)			(ref)		
Number of metastatic sites	1	(ref)			(ref)		
	2	1.05	0.66–1.67	0.84	1.31	0.82–2.10	0.27
	>2	1.23	0.77–1.96	0.40	1.51	0.95–2.40	0.09
Prior chemotherapy	F	(ref)			(ref)		
	F + Oxa	0.99	0.52–1.92	0.99	2.50	1.25–5.00	0.009
	F + Oxa + Bev	1.91	0.52–6.96	0.33	3.53	0.94–13.2	0.06
Prior radiotherapy	Yes	0.71	0.29–1.75	0.46	0.85	0.35–2.10	0.73
	No	(ref)			(ref)		
Liver metastases	Yes	0.88	0.57–1.37	0.58	1.18	0.76–1.82	0.64
	No	(ref)			(ref)		
Lung metastases	Yes	1.09	0.73–1.61	0.67	1.50	1.01–2.21	0.044
	No	(ref)			(ref)		
*TOP1* CN (CEN-20)	Per 1 unit increase	0.99	0.90–1.10	0.88	0.98	0.89–1.08	0.72
*TOP1 CN (CEN-20)*	>median (4.46)	0.95	0.65–1.40	0.79	0.92	0.62–1.35	0.66
	≤ median (4,46)	(ref)			(ref)		
*TOP1*/CEN-20 ratio	Per 1 unit increase	0.99	0.56–1.74	0.97	1.02	0.61–1.70	0.95
*TOP1*/CEN-2 ratio	Per 1 unit increase	1.10	0.85–1.35	0.58	1.00	0.79–1.27	0.98
*TOP1*/CEN-20 ratio	<1.5 (ref)						
	≥1.5	0.73	0.47–1.13	0.16	0.75	0.48–1.17	0.21

*Abbreviations*: *F* Fluorouracil (5-FU), *Oxa* Oxaliplatin, *Bev* Bevacizumab, *HR* Hazard ratio, *CI* Confidence interval

^a^Unknowns were not included in the shown proportions or test for distribution differences

**Fig. 4:**
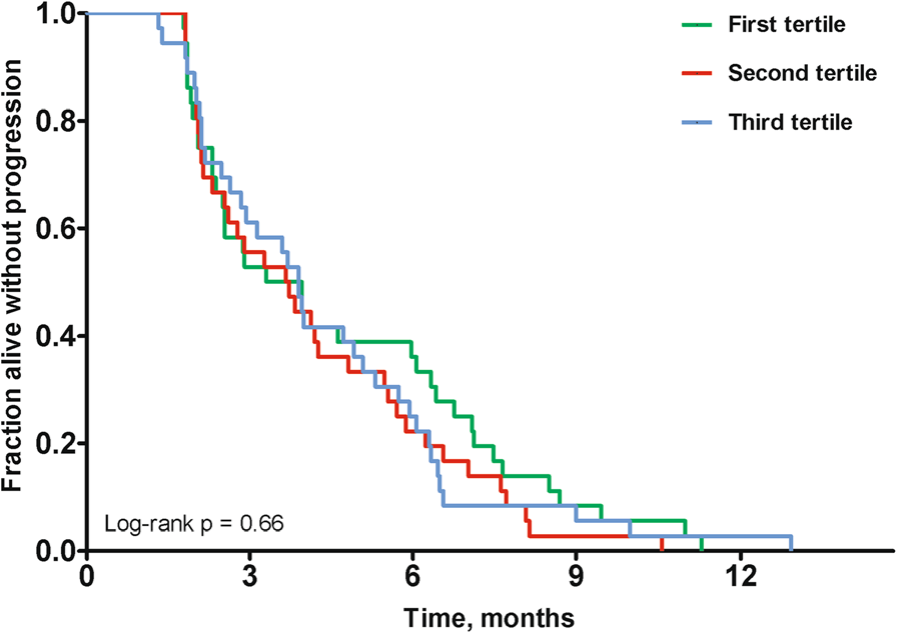
Kaplan-Meier plot for progression-free survival as a function of *TOP1* gene copy number. Patients were divided into tertiles by *TOP1* copy number

We did not find any associations between the dichotomized version of *TOP1* CN and PFS and OS as HRs were 0.95 (CI 0.65–1.40; *p* = 0.79) and 0.92 (CI 0.62–1.35; *p* = 0.66), respectively. We also tested PFS as a function of *TOP1* CN divided into tertiles and found no significant association, log-rank *p* = 0.66 (Fig. 4).

According to the definitions used, 9% had an amplified tumor (*TOP1*/CEN-20 ratio ≥ 2 and *TOP1*/CEN-2 ratio ≥ 2), 44% had a tumor harboring a q-20 polysomy (*TOP1*/CEN-20 ratio ≤ 2 and *TOP1*/CEN-2 ratio ≥ 2), and 46% had a tumor that was neither amplified nor a polysomy. Patients with tumors classified as harboring a *TOP1* amplification did not have improved PFS: HR 1.71 (CI 0.88–3.32; *p* = 0.12) or OS: HR 1.32 (CI 0.69–2.54; *p* = 0.41). Neither did patients with tumors classified as harboring q-20 *TOP1* polysomy: PFS HR 0.94 (CI 0.64–1.38; *p* = 0.75) and OS HR 0.71 (CI 0.48–1.05; *p* = 0.08). There were no significant associations between *TOP1* amplification or q-20 *TOP1* polysomy and objective response.

No multivariate analysis was performed since the variables tested were not significant in the univariate analyses.

## Discussion

This study is the first to report the CN of *TOP1* and the ratios of *TOP1*/CEN-20 and *TOP1*/CEN-2 in CRC patients and their relation to objective response to irinotecan in a metastatic setting. We added a probe for CEN-2 to discriminate between an increase in *TOP1* CN due to mechanisms localized to chromosome 20 and an increase caused by increased ploidy level. We chose chromosome 2 because this chromosome has been reported to undergo fewer alterations when compared to other chromosomes in cancer specimens [24]. In accordance with this, a recent study investigating *TOP1* CN aberrations, considered CEN-20 CN as an inappropriate marker for cellular ploidy based on the frequent gain of chromosome 20 or 20q [21].

*TOP1* cut-off values were chosen based on the median value. This decision was partly based on the results from a similar study [25] and partly to obtain two equally sized groups. In our study, we found a higher median gene CN for *TOP1* (4.46) than reported in the study by Nygaard et al. (3.6) [25]. A plausible explanation for this discrepancy could be the difference in number of cells counted. In our study we counted a minimum of 60 signals of *TOP1* CN for each patient giving a median of 14.5 and a range of 8–40 counted nuclei. This is less than the 60 nuclei counted by Nygaard et al. One could argue that since we selected the nuclei with the best quality signals for counting we also tended to select the nuclei with the highest CNs. Despite the difference in methods, our study showed the same results as Nygaard et al. concerning the association with objective response, PFS, and OS.

Previously, a positive correlation between the *TOP1* CN and in vitro sensitivity to SN-38 was reported in CRC cell lines and this was also demonstrated for the *TOP1*/CEN-20 ratio. Notably, the correlation for the *TOP1* CN was superior to the *TOP1*/CEN-20 ratio, which is consistent with the data in our study [18]. Our results demonstrated a borderline significant association between *TOP1* CN and objective response (*p* = 0.08), which is in accordance with the results by Nygaard et al. (*p* = 0.07) [19]. However, this result was not further supported as there were no association between *TOP1* CN and PFS. Regarding the ratios of *TOP1*/CEN-20 or *TOP1*/CEN-2 and response to irinotecan we found no associations to irinotecan response. However, the *TOP1* CNs was significantly correlated to the CEN-20 CNs which may mask *TOP1* amplification. Collectively, these findings could support the use of gene CN only instead of a gene/CEN ratio, which is further supported from the clinically implemented human epidermal receptor-2 (*HER2*) FISH analysis where only *HER-2* CN can be reported without the ratio in cases with coamplification of *HER-2* and CEN-17 [26].

The patients in this study constituted a selected group with a good prognosis as they were identified from a national cohort characterized by all patients having received third line treatment (irinotecan + cetuximab). Besides a long median OS (16.4 months) as could be expected in this selected group, the clinical data in this study corresponded to what have previously been reported from studies including irinotecan second-line monotherapy with RR of 10% [27]. With 108 patients and RR of only 9% we have limited power to detect a predictive value of *TOP1* as a biomarker for response. For that reason, we also investigated clinical benefit rate.

A major obstacle for obtaining a high level of evidence for predictive biomarkers is the lack of well-defined cut-off values for overexpression and gene amplification. Even for a well-established biomarker such as *HER-2* in breast cancer, cut-off values are still discussed and have changed over time.

The level of Top1 protein expression could be useful as a predictive biomarker for the response to Top1 targeting chemotherapy. A number of studies have investigated this possibility in patients having CRC, but with conflicting results [10–14]. These inconsistent results may partly be explained by the fact that immunohistochemistry (IHC) requires validated antibodies as well as standardized protocols, both of which have been difficult to obtain for Top1. Accordingly, Top1 IHC has not yet reached the required level for clinical implementation.

Another aspect is the different DNA repair mechanisms activated upon DNA damage which may in part explain why *TOP1* CN alone could not predict response. A study reported that the expression of aprataxin, a histidine triad domain superfamily protein involved in DNA repair, in 30 CRC cell lines was correlated with sensitivity to irinotecan [28]. Another repair pathway, the tyrosyl-DNA-phosphodiesterase I (TDP1) was investigated in clinical CRC samples and cell lines. The authors reported that TDP1 is involved in the resolution of DNA damage associated with Top1 poisons and that the protein expression levels of TDP1 or Top 1 alone were not associated with sensitivity to irinotecan [29].

A robust correlation between *TOP1* CN status, gene expression level, protein expression level and -activity in cancer cell lines [9, 30] and cancer tissues [31, 32] has been reported. Accordingly, the *TOP1* CN may be a useful “proxy” predictive biomarker for Top1 targeting drugs. One concern in this study is whether the *TOP1* CN determined in primary tumors correspond to the CN in the treated metastases. In our study primary tumors were analyzed in order to test our hypothesis. However, the majority of the treated tumor volumes in these patients consisted of metastases. Conflicting results have been reported regarding the concordance between primary tumor and metastases. Two studies reported a significantly higher Top1 expression in the metastases than in the corresponding primary tumor [33] [34]. Yet, other studies investigating *TOP1 mRNA* [35] and Top1 protein levels [36] have reported concordance between primary tumors and metastases. Another concern is the impact of prior treatments as it may change the biologic profile between the primary and recurrent tumors. An ideal design to overcome the difficulties mentioned above would be to assess *TOP1* CN in the metastasis from mCRC prior to first-line irinotecan-containing therapy.

## Conclusions

We verified a borderline significant association between increasing *TOP1* CN and objective response to irinotecan monotherapy in mCRC patients as previously reported. Applying CEN-20 and CEN-2, to *TOP1*, did not provide further information to assist a biomarker driven patient stratification, suggesting that other biomarkers should be paired with *TOP1* CN.
